# Relationships between determinants of adjuvant endocrine therapy adherence in breast cancer

**DOI:** 10.1186/s12905-018-0522-3

**Published:** 2018-03-19

**Authors:** Joo Yun Lee, Yul Ha Min

**Affiliations:** College of Nursing, Gachon University, 191 Hambakmeo-ro, Yeonsu-gu, Incheon, 21936 South Korea

**Keywords:** Medication adherence, Breast cancer, Hormone therapy, Predisposing factor

## Abstract

**Background:**

Interventions that promote adjuvant endocrine therapy (AET) adherence are critical to improve breast cancer survival. The development of interventions would benefit from a better understanding of the reasons for adherence and the causal relationships of determinants using theoretical or model approaches. The aim of the present study was to identify reasons for AET adherence in breast cancer patients with sequential relationships and inter-relationships.

**Methods:**

A total of 210 participants with estrogen receptor positive breast cancer who received AET completed a questionnaire assessing demographic/medical, psychological, and endocrine therapy (ET)-specific factors. A descriptive analysis was performed to identify meaningful variables. Selected variables were subjected to hierarchical regression and path analyses. The path model was tested and modified based on the research framework and the results of regression weights and model fit.

**Results:**

Analysis of sequential effects showed that ET-specific factors contributed the largest proportion of variance (13.4%) to predict AET adherence, followed by psychological factors (4.6%) and demographic/medical factors (3.1%). Analysis of inter-relationships showed that demographic/medical factors such as AET regimen type and cancer stage have direct effects on AET adherence, whereas psychological factors contribute indirectly through the mediating effects of ET-specific factors.

**Conclusion:**

Assessments and interventions that encompass the patient’s medication beliefs, self-efficacy, and depression are needed to promote AET adherence.

## Background

To reduce the risk of recurrence and mortality in women diagnosed with hormone receptor positive breast cancer, current guidelines generally recommend 10 years of adjuvant endocrine therapy (AET) [1]. Despite the efficacy of AET, adherence rates range from 41% to 72% when measured over 4 years [2]. Early discontinuation or non-adherence to AET is associated with increased mortality [3]. Therefore, interventions aimed at improving AET adherence are critical to increase breast cancer survival rates.

The development of patient-centered intervention strategies is important to promote patient adherence to treatment. Identifying the determinants of adherence and explaining the causal relationship of determinants using theoretical or model-based methods may help understand the reasons for adherence and thus the development of patient-centered intervention approaches.

Two analytical methods were proposed to examine the relationships between variables based on current models [4]. Hierarchical regression analysis is useful to assess the effect of determinants on predicting the outcome in a sequential manner. Path analysis is well suited to evaluate the inter-relationships between determinants and their direct and indirect effects on the outcome.

A study that examined the hierarchical effects of determinants for AET adherence suggested the existence of three levels of determinants, namely, demographic/medical factors, psychological factors, and endocrine therapy (ET)-specific factors [5]. Demographic/medical factors include variables such as age, education, employment status, type of AET, and operation type [5–7]. Depression, anxiety, and perceived physical symptoms [8–10] could be categorized into psychological factors. ET-specific factors include necessity-concerns beliefs on medication and self-efficacy with regard to AET [8, 11].

However, the inter-relationships between AET adherence factors have not been investigated yet. The patient adherence model hypothesized patient-related determinants of adherence in a more comprehensive way to determine inter-relationships between AET adherence factors [12]. According to the determinants of the patient adherence model, which examined for dyspepsia medication adherence, demographic/medical and psychological factors could contribute to necessity-concerns beliefs on medication and self-efficacy. These associations could be applied to the analysis of patients who are prescribed AET. The design of patient centered interventions to improve adherence could be considerably facilitated by determining the reasons for adherence from both sequential relationships and inter-relationships.

**Fig. 1:**
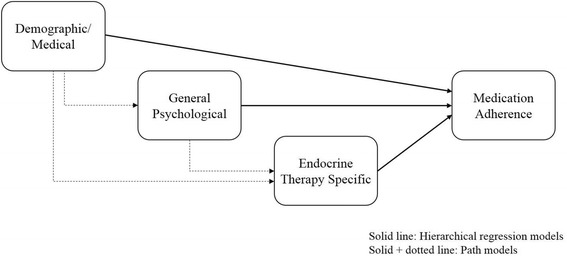
Research framework of the study

The purpose of the present study was to identify reasons for adherence with sequential relationships and inter-relationships. Based on previous studies [8, 12], we proposed two hypotheses within the research framework (Fig. 1). First, three levels of determinants would affect AET adherence sequentially. Second, demographic/medical and psychological factors could affect ET-specific factors, and determinants could contribute indirectly to ET therapy adherence. We identified the level of determinants that are predictable and the level of determinants with a high impact using hierarchical regression analysis. In addition, the path model from the research framework was evaluated and modified to identify the optimal model to explain AET adherence using path analysis.

## Methods

### Design and participants

A cross-sectional design was used in the present study. Participants were recruited from the outpatient clinic of Asan Medical Center, a University-affiliated hospital in South Korea, between July 15 and August 24, 2016. All patients with histologically confirmed breast cancer were consecutively screened for eligibility. The selection criteria were as follows: age ≥ 20 years; a completed regimen of neoadjuvant or adjuvant chemotherapy; a diagnosis of stage 0, 1, 2, or 3 breast cancer; no history of psychiatric or neurologic illness; no recurrence or presence of secondary breast cancer; and ongoing AET for breast cancer at the time of the visit. After screening for eligibility, 400 eligible patients were asked to participate by a research nurse and 254 patients consented. Out of 254 patients, 44 patients were excluded because they had not responded entirely to all of the questions in the questionnaire. In total, data from 210 patients (52.5%) were used in the analyses. All participants completed written informed consent forms approved by the Institutional Review Board (no. 2016–0351).

### Study variables

#### Adherence

Adherence to AET therapy was assessed using the Morisky Medication Adherence Scale-8 (MMAS-8) questionnaire, which uses an 8-item scale [13–15]. Scores obtained on the MMAS-8 range from 0 to 8, with higher scores indicating higher adherence. Permission to use the validated Korean MMAS-8 was obtained from Dr. Morisky.

#### Demographic/medical level variables

Demographic variables included age, marital status, menopausal status, employment status, educational level, and family history of cancer. Medical variables included the type of AET, time from the start of AET, type of surgery, cancer stage, and completion of chemotherapy or radiotherapy. Demographic and medical variable data of the participants were retrieved using the electronic medical record (EMR) system with the consent of patients.

#### Psychological level variables

Menopause-related symptoms were measured using the 11-item Menopause Rating Scale (MRS) [16]. Patients responded to 11 items on a 5-point Likert scale ranging from 0 (no complaints) to 4 (severe symptoms). The Korean version of the MRS scale was obtained from the MRS network (http://www.menopause-rating-scale.info) after obtaining permission from Dr. Heinemann.

Symptoms of depression were measured using the 20-item Center for Epidemiological Studies-Depression Scale (CES-D) developed by the National Institute of Mental Health. The Korean version of the CES-D that was specifically developed for Korean populations was used [17, 18]. Patients were asked to indicate their responses using a 4-point Likert scale (from 0 = rarely or never to 3 = almost all or all of the time). Scores ranged from 0 to 60, with higher scores reflecting more severe depression symptoms.

The short version of the Fear of Progression Questionnaire (FoP-Q-SF), a 12-item scale [19, 20], was used to measure the degree of fear of disease progression. The FoP-Q-SF items were scored on a 5-point Likert scale ranging from 1 (never) to 5 (very often), with higher values indicating higher levels of anxiety.

#### ET-specific level variables

The beliefs about AET were assessed using the Beliefs about Medicines Questionnaire (BMQ)-Specific. The questionnaire includes five queries that address the necessity of prescribed medications (necessity beliefs) and five queries that address concerns regarding potentially adverse consequences while taking the medications (concern beliefs). Each subscale score ranges from 5 to 25, with higher BMQ-necessity scores meaning higher perceived medication necessity, and higher BMQ-concern scores indicating higher perceived medication concerns. The BMQ-Specific was used with the permission of the questionnaire developer, Prof. Horne [21]. The original English version of the BMQ-Specific was translated to Korean and then translated back to English by two independent clinical experts and a native English speaker to ensure semantic and structural equivalency [22].

Participants responded to questions of the Korean version of the general self-efficacy (GSE) scale [23], which was originally developed in Germany by Matthias Jerusalem and Ralf Schwarzer [24]. The GSE is a 10-item psychometric scale ranging from 0 to 40, with higher scores representing a higher level of self-efficacy.

### Analysis

A descriptive analysis of the demographic/medical, psychological, and ET-specific characteristics was performed. The correlation between these independent variables and the MMAS was determined to identify meaningful variables that could be subjected to hierarchical regression and path analyses. Correlation analysis was used for continuous variables, and the t or F test was used for categorical variables.

To identify the sequential effects of the three levels of determinants, selected variable sets were entered into a hierarchical regression analysis in three steps: demographic/medical variables, psychological variables, and ET-specific variables.

The inter-relationships between selected demographic/medical, psychological, and ET-specific variables were investigated. The first path model was designed in accordance with the hypothesis of three levels of determinants. The path model was tested and modified by adding and removing a path based on the research framework and the results of regression weights and model fit [4, 25]. The model fit was compared using the Chi-square, normed fit index (NFI), comparative fit index (CFI), and root mean square error of approximation (RMSEA) methods. Standardized coefficients and calculated direct, indirect, and total effects were reported. IBM SPSS 23 and AMOS 21 were used for analysis.

## Results

### Characteristics according to the three levels

A total of 210 patients were included in the study. The mean age of patients was 50.31 years (SD = 9.03, range = 25–76), and most of them were married (90.4%). There were 19 (9.0%) participants who had a family history of breast cancer, and 83 (39.5%) participants who had a family history of other cancers. Most participants had conservational surgery (87.1%), and received selective estrogen receptor modulators (SERMs) (77.6%). The mean time on AET was 26.75 months (Table 1).

**Table 1:** Descriptive analysis of variables according to the three levels

		Mean (SD) or n (%)	Endocrine therapy adherence (MMAS)			
			Mean (SD)	r	t or F	p
Total MMAS score			6.36 (1.65)			
Demographic/medical						
Age		50.31 (9.03)		.136		.050
Education level	College above	84 (40.8)	6.12 (1.64)		1.64	.197
	High	90 (43.7)	6.50 (1.56)			
	Below high	32 (15.5)	6.63 (1.89)			
Marital status	No	20 (9.6)	6.20 (1.35)		−.473	.637
	Yes	189 (90.4)	6.38 (1.68)			
Employment status	No	117 (56.5)	6.48 (1.57)		1.103	.271
	Yes	90 (43.5)	6.23 (1.75)			
FAM HX of breast cancer	No	191 (91.0)	6.36 (1.69)		−.028	.975
	Yes	19 (9.0)	6.37 (1.16)			
FAM HX of other cancer	No	127 (60.5)	6.44 (1.64)		.856	.393
	Yes	83 (39.5)	6.24 (1.66)			
Cancer stage	Stage 1–3	184 (87.6)	6.47 (1.59)		2.554	.011
	Stage 0	26 (12.4)	5.60 (1.89)			
Node meta	Negative	145 (69.0)	6.27 (1.68)		−1.221	.223
	Positive	65 (31.0)	6.57 (1.57)			
Type of surgery	Mastectomy	27 (12.9)	6.71 (1.37)		1.200	.232
	Conservation	183 (87.1)	6.31 (1.68)			
RT	No	33 (15.7)	6.39 (1.60)		−.135	.893
	Yes	177 (84.3)	6.35 (1.66)			
CT	No	110 (52.4)	6.28 (1.68)		.705	.481
	Yes	100 (47.6)	6.44 (1.61)			
AET regimen	AI	47 (22.4)	6.79 (1.54)		2.068	.040
	SERM	163 (77.6)	6.23 (1.66)			
Months on ET		26.75 (19.79)		−.012		.858
FSH level		21.83 (24.31)		.160		.100
General psychological						
Menopause-related symptom (0–44)		13.32 (7.71)		−.087		.214
Depression (0–60)		15.56 (9.83)		−.204		.004
Fear of progression (12–60)		31.60 (10.79)		−.053		.458
Endocrine therapy-specific						
Beliefs about AET (10–50)		24.50 (6.47)		−.005		.945
Necessity beliefs (5–25)		13.83 (4.17)		.235		.001
Concern beliefs (5–25)		10.68 (4.10)		−.247		.000
Self-efficacy (0–40)		28.99 (4.41)		.187		.007

*MMAS* Morisky Medication Adherence Scale, *FAM HX* family history, *RT* radiotherapy, *CT* chemotherapy, *AI* aromatase inhibitor, *SERM* selective estrogen receptor modulator, *ET* endocrine therapy, *FSH* follicle-stimulating hormone. Permission for MMAS-8 was obtained from Dr. Morisky and MMAS Research. The Morisky Widget, MMAS-8 and MMAS-4, are protected by US and International Trademark and Copyright laws. Permission for use is required. A license agreement is available from: MMAS Research LLC 14725 NE 20th St. Bellevue WA 98007

The mean score for AET therapy adherence was 6.36 (SD = 1.65, range = 0.3–8.0). When the mean score was compared to the cutoff for adherence [13], there were 54 (25.7%) highly adherent patients with a score of 8 on the scale, 79 (37.6%) medium adherers with a score of 6 or 7, and 77 (36.7%) low adherers with a score of below 6.

At the demographic/medical level, participants who had invasive cancer (stage 1–3) (*t* = 2.554, *p* = 0.011) and received aromatase inhibitors (AI) (*t* = 2.068, *p* = 0.040) had higher MMAS scores.

At the psychological level, a lower CES-D score indicating a lower depression level was significantly related to a higher MMAS score. Patient with higher necessity beliefs and self-efficacy and lower concerns beliefs tended to have higher MMAS scores at the ET-specific level.

### Sequential effects of determinants according to the three levels

Based on the three levels of determinants, cancer stage, AET regimen type, depression, necessity belief, concerns belief, and self-efficacy were identified as significant variables. To compare the effects of the three levels of determinants, MMAS scores were regressed to the variables using hierarchical regression analysis. The first model contained only cancer stage and AET regimen type and accounted for 3.1% of the variance in ET therapy adherence [F (2,195) = 3.087, *p* = 0.048]. The second model included the depression variable and accounted for 7.7% of the variance [F (3,194) = 5.418, *p* = 0.001]. The change of R-square was significant (*p* = 0.002). The final model included concerns belief, necessity belief, and self-efficacy and accounted for 21.1% of the variance [F (6,191) = 8.488, *p* < 0.001]. The change of R-square was also significant (p < 0.001). This can be interpreted as ET-specific factors explaining the largest proportion (13.4%) of variance. In the final model, AET regimen type (β = 0.147, *p* = 0.027), concerns belief (β = − 0.287, p < 0.001), necessity belief (β = 0.284, p < 0.001), and self-efficacy (β = 0.142, *p* = 0.045) contributed to adherence significantly. The depression and concerns belief scores had negative effects on adherence in accordance with Pearson’s coefficients (Table 2).

**Table 2:** Sequential effects of determinants according to the three levels

		Model 1				Model 2				Model 3			
		B	S.E.	β	t	B	S.E.	β	t	B	S.E.	β	t
Demographic/ medical	AET regimen	.420	.276	.110	1.520	.421	.270	.110	1.559	.564	.254	.147	2.225^**^
	Cancer stage	.576	.357	.116	1.613	.659	.350	.133	1.880	.604	.327	.122	1.845
Psychological	Depression					−.036	.011	−.217	−3.131^**^	−.009	.013	−.055	−.690
ET-specific	Necessity beliefs									.111	.026	.284	4.282^**^
	Concern beliefs									−.113	.030	−.287	−3.755^**^
	Self-efficacy									.053	.026	.142	2.018^**^
R^2^ (Adjusted R^2^)		.031 (.021)				.077 (.063)				.211 (.186)			
F for R change						9.802^**^				10.742^**^			

Significance of F change *p < .05, **p < .01

### Inter-relationships between determinants

**Fig. 2:**
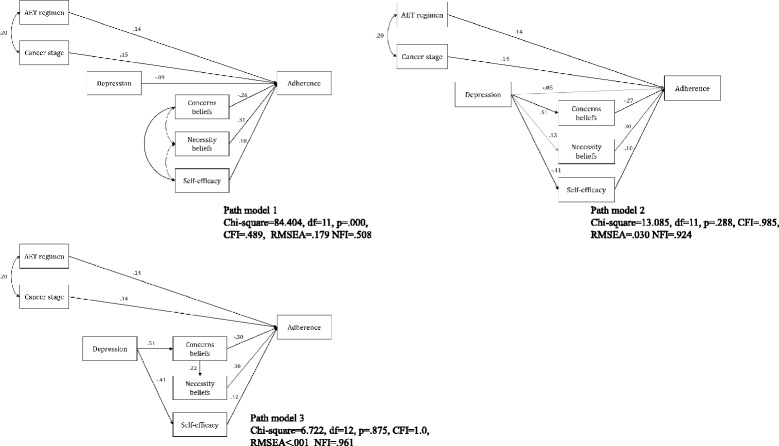
Path model explaining the effects of determinants

The first path model (Fig. 2-path model 1) was tested, and the model fit indices were unacceptable, showing a significant Chi-square value [χ^2^ (df = 11, *n* = 210) = 84.404, *p* < 0.001, CFI = 0.489, RMSEA = 0.179, NFI = 0.508]. The paths were modified according to the statistical analysis results and a review of the literature. The analysis showed that depression had a stronger relation to concerns beliefs (*r* = 0.506, *p* < 0.001) and self-efficacy (*r* = − 0.397, p < 0.001) than to ET adherence (*r* = − 0.204, *p* = 0.004), and it had a weak relationship with necessity belief (*r* = 0.139, *p* = 0.051). Demographic/medical factors including AET regimen type and cancer stage were not related to psychological and ET-specific factors. After changing the paths and testing the models (Fig. 2-path model 2), a final model was obtained (Fig. 2-path model 3). The model fit indices of the final path model indicated a good fit to the data [χ^2^ (df = 12, *n* = 210) = 6.722, *p* = 0.875, CFI = 1.0, RMSEA < 0.001, NFI = 0.961]. All the direct path coefficients were significant at the 0.05 level. The standardized path coefficients linking depression to concerns beliefs and depression to self-efficacy were relatively large (Fig. 2-b). The total effect was largest in necessity belief to ET adherence (Table 3).

**Table 3:** Decomposition of effects from the path model

	Unstandardized			Standardized		
	Direct	Indirect	Total	Direct	Indirect	Total
AET regimen	.566		.566	.143		.143
Cancer stage	.709		.709	.142		.142
Depression		−.028	−.028		−.167	−.167
Necessity beliefs	.120		.120	.302		.302
Concern beliefs	−.119	.027	−.092	−.295	.067	−.228
Self-efficacy	.045		.045	.120		.120

## Discussion

In the present study, determinants of AET adherence were examined regarding sequential relationships and inter-relationships.

Univariate analysis showed that MMAS scores differed significantly according to AET regimen type and cancer stage. Also, MMAS scores correlated with depression, beliefs on medication and self-efficacy. Thus, these variables were selected for further multivariate analyses.

Analysis of the sequential effects of the three factors showed that ET-specific factors were responsible for the largest variance (13.4%) to predict AET adherence, followed by psychological factors (4.6%) and demographic/medical factors (3.1%). Additional entry of each factor increased R^2^ significantly, resulting in a final regression model that accounted for 21.1% of the variance in adherence. This result was consistent with those of previous studies [8, 26] and carries implications for intervention strategies based on the three levels of factors. In detail, depression was statistically significant in model 2, which includes only demographic/medical and psychological factors. However when ET-specific factors were added, depression was no longer significant. Therefore, additional analyses of the direct and indirect effects of the determinants were performed.

There are few reports on the effect of AET regimen type on adherence. One study reported that AET regimen type is a determinant for adherence [6, 27]. The present data did not show an association between AET regimen type and psychological variables or ET-specific variables. However, among the demographic/medical factors, AET regimen type had a significant relationship with age (*t* = 10.842, *p* ≤ 0.001), FSH level (*t* = 12.488, *p* < 0.001), and node metastasis (χ^2^ = 7.123, *p* = 0.008). Similar to AET regimen type, cancer stage was not related to psychological variables or ET-specific variables in the present study, whereas a relationship with type of surgery (χ^2^ = 4.378, *p* = 0.036) was identified from the data. AET regimen type and cancer stage seemed to show a relationship with other demographic/medical factors that affect AET adherence. Additional studies could be suggested to explain these relationships.

The demographic/medical level findings indicated that they have direct effects on AET adherence, rather than indirect effects as predisposing factors of psychological or ET-specific level factors. Variables identified at this level draw attention because they are non-modifiable risk factors and specific characteristics of breast cancer patients.

Depression is a known determinant of medication adherence. However, the mechanism underlying the relationship between depression and medication adherence is not well understood. A previous study suggested that participants who are anxious or depressive in the early stages of treatment are likely to be motivated to take medication [9]. By contrast, the results of our study indicated that highly depressive patients tend to be non-adherent, and this result is consistent with those of other previous studies [8, 28, 29]. We have tried to explain this result as an indirect effect of depression with ET-specific factors, since the effects of self-efficacy and medication beliefs on medication adherence are known. High self-efficacy, high positive beliefs on medication, and low negative beliefs on medication are more likely to lead to adherence to prescribed medications [5, 11, 30]. Several studies which focused on self-efficacy and medication beliefs as mediators linking depression and medication adherence. A Schoenthaler, G Ogedegbe and JP Allegrante [31] found that self-efficacy played a mediating role between depression and medication adherence in hypertensive patients. ME Hilliard, MN Eakin, B Borrelli, A Green and KA Riekert [32] reported that medication beliefs were the mediators in cystic fibrosis patients. The present study supports the idea that depression contributed to ET-specific factors. Higher depression was statistically related to higher concerns beliefs, lower self-efficacy, and consequently lower adherence to AET. Although necessity beliefs were not affected by depression, there was a significant association between concerns beliefs and necessity beliefs. The direct effect of depression on medication adherence was not significant when the mediating variables were included in the model. The mechanism underlying the relationship between depression and medication adherence can be explained using ET-specific factors as mediating factors including self-efficacy and medication beliefs. These results suggest that assessments and interventions need to consider the patient’s medication beliefs, self-efficacy, and depression to promote AET adherence. Interventions could vary according to the associated depression. Interventions for medication beliefs and self-efficacy are usually delivered by educating and counseling, whereas treatments for depression could include a pharmacological approach [28]. In addition, the efficacy of depression self-care intervention for increasing self-efficacy was reported previously [33].

Understanding the mechanism underlying medication adherence is important to increase the adherence rate and promote a positive health outcome, and there are ongoing efforts to explain this mechanism. To the best of our knowledge, these efforts have mainly focused on identifying determinants of AET adherence. Few studies have addressed the relationships among the factors [6, 30]. We designed a research framework based on the hierarchical model of AET adherence determinants [8] and a determinants of adherence model that was developed for dyspepsia medication [12]. Our data showed that associations between psychological factors and ET-specific factors were well fitted to the research framework, whereas demographic/medical factors showed discrepancies with the research framework. Finally, a path model was generated to explain the adherence to AET using demographic/medical, psychological, and ET-specific factors. These results could be used during the development of patient-centered interventions. Tailored interventions could be planned depending on the regimen and cancer stage. Also, the patient’s medication beliefs and self-efficacy should be assessed in combination with depression to implement individualized interventions.

The cross-sectional study design of the present study limits the interpretation of the results as causal relationships. To explain the causal relationships with evidence, we hypothesized relationships based on previous research and evaluated by path analysis. Additional studies are needed to confirm these relationships. Although our sample was from one of the top five major cancer treating about 15% of all breast cancer patients in Korea and the patients were recruited by five different surgeons, bias could have occurred because the participants were recruited in a single tertiary hospital setting and the reason for the rejection of non-participants was not analyzed. More analyses assuring the representativeness of the study population will be needed to decrease the bias. Lastly, the AET adherence data were collected using subjective measurements based on self-reporting. The poor ability of self-reports to measure adherence is well known [34, 35]. Although we used MMAS-8, which has shown reliability and validity, further studies using objective measurements will be necessary.

## Conclusion

The present study investigated the determinants of AET adherence as AET regimen type and cancer stage at the demographic/medical level, depression at the psychological level, and belief and self-efficacy at the ET-specific level. Despite the direct effects of AET regimen type and cancer stage on AET adherence, depression contributes as an ET-specific factor such as concerns beliefs and self-efficacy. To promote AET adherence, assessments and interventions should consider the patient’s medication beliefs, self-efficacy, and depression level.

## Abbreviations

AET: Adjuvant endocrine therapy
AI: Aromatase inhibitor
BMQ: Beliefs about Medicines Questionnaire
CES-D: Center for Epidemiological Studies-Depression Scale
CFI: Comparative fit index
CT: Chemotherapy
EMR: Electronic medical record
ET: Endocrine therapy
FAM HX: Family history
FoP-Q-SF: Fear of Progression Questionnaire
FSH: Follicle-stimulating hormone
GSE: General self-efficacy
MMAS: Morisky Medication Adherence Scale
MRS: Menopause Rating Scale
NFI: Normed fit index
RMSEA: Root mean square error of approximation
RT: Radiotherapy
SERM: Selective estrogen receptor modulator
